# Techno-Functional, Rheological, and Physico-Chemical Properties of Gelatin Capsule By-Product for Future Functional Food Ingredients

**DOI:** 10.3390/foods14071279

**Published:** 2025-04-07

**Authors:** Sasina Sanprasert, Pudthaya Kumnerdsiri, Anusorn Seubsai, Piyangkun Lueangjaroenkit, Jaksuma Pongsetkul, Sylvia Indriani, Tanyamon Petcharat, Samart Sai-ut, Kanrawee Hunsakul, Utthapon Issara, Subhash V. Pawde, Saroat Rawdkuen, Thomas Karbowiak, Young Hoon Jung, Passakorn Kingwascharapong

**Affiliations:** 1 Department of Fishery Products, Faculty of Fisheries, Kasetsart University, Bangkok 10900, Thailand; sasina.sanp@ku.th (S.S.); pudthaya.ku@ku.th (P.K.); 2 Department of Chemical Engineering, Faculty of Engineering, Kasetsart University, Bangkok 10900, Thailand; fengasn@ku.ac.th; 3 Department of Microbiology, Faculty of Science, Kasetsart University, Bangkok 10900, Thailand; piyangkun.lu@ku.th; 4 School of Animal Technology and Innovation, Institute of Agricultural Technology, Suranaree University of Technology, Nakhon Ratchasima 30000, Thailand; jaksuma@sut.ac.th (J.P.); indrianisylvia@gmail.com (S.I.); 5 Professional Culinary Arts Program, School of Management, Walailak University, Thasala, Nakhon Si Thammarat 80161, Thailand; tanyamon.pet@gmail.com; 6 Department of Food Science, Faculty of Science, Burapha University, Chonburi 20131, Thailand; samarts@go.buu.ac.th; 7 Division of Agro-Industrial Product Development, Faculty of Science and Technology, Rajamangala University of Technology Tawan-ok, Chonburi 22210, Thailand; kanrawee.h@hotmail.com; 8 Division of Food Science and Technology Management, Faculty of Science and Technology, Rajamangala University of Technology Thanyaburi, Pathum Thani 12110, Thailand; utthapon_i@rmutt.ac.th; 9 Unit of Innovative Food Packaging and Biomaterials, School of Agro-Industry, Mae Fah Luang University, Chiang Rai 57100, Thailand; pawdesubhash333@gmail.com; 10 Université Bourgogne Europe, Institut Agro, INRAE, UMR PAM, F-21000 Dijon, France; thomas.karbowiak@institut-agro.fr; 11 School of Food Science and Biotechnology, College of Agriculture and Life Sciences, Kyungpook National University, Daegu 41566, Republic of Korea; younghoonjung@knu.ac.kr

**Keywords:** gelatin, capsule waste, maltodextrin, techno-functional, future food ingredients

## Abstract

The utilization of gelatin capsule waste (GCW) poses a challenge for the industry. This study investigates its potential as a functional food ingredient by evaluating the physico-chemical, rheological, and techno-functional properties of gelatin capsule waste powder (GCWP). To achieve this, the gelatin capsule waste (GCW) was mixed with maltodextrin at varying ratios (1:1, 1:2, 1:3, 1:4, and 1:5) and subjected to spray drying. The findings highlight maltodextrin’s crucial role in stabilizing the drying process, reducing stickiness, and enhancing handling and storage properties. All the obtained GCWP samples appeared light white and had a slightly sticky texture. The 1:5 (*w*/*w*) GCW-to-maltodextrin ratio produced the highest powder recovery with minimal stickiness, indicating enhanced drying efficiency. Increasing maltodextrin reduced gel strength, texture, and foaming properties while raising the glass transition temperature. The FTIR analysis indicated a decline in protein–protein interactions and increased polysaccharide interactions at higher maltodextrin levels. The rheological analysis demonstrated lower elastic and loss moduli with increased maltodextrin, affecting GCWP’s structural behavior. For overall properties, the GCW mixed with maltodextrin at a 1:1 ratio (GCW-1M) is recommended for future applications, particularly for its gelling characteristics. The GCW-1M, being rich in amino acids, demonstrates its potential as a functional food ingredient. However, certain properties, such as gel strength and powder stability (hygroscopicity and stickiness), require further optimization to enhance its industrial applicability as a functional food ingredient.

## 1. Introduction

The future of food production is increasingly focused on functional food ingredients that provide health benefits beyond basic nutrition. This shift reflects a growing demand for nutrient-dense, sustainable, and scientifically validated functional foods that support overall well-being. Functional food ingredients are bioactive compounds or naturally occurring components that enhance health by improving digestion, boosting immunity, or reducing disease risk. These ingredients include proteins, bioactive peptides, dietary fibers, probiotics, and plant-based antioxidants, which are commonly incorporated into food products to deliver targeted health benefits. The global functional food market is projected to reach over USD 275 billion by 2025, driven by consumer preferences for health-promoting foods [1]. Notably, gelatin and its derivatives play a crucial role in this market due to their functional properties, including gelation, emulsification, and encapsulation capabilities. Simultaneously, the nutraceutical and pharmaceutical industries are experiencing a rising demand for capsule shells due to their efficiency in encapsulating medicines and active ingredients [2,3]. Kalmer, et al. [4] reported that the global capsule shell market is predicted to be worth around USD 2.9 billion by 2026. For centuries, gelatin has been most commonly used to produce shell material according to desired properties, such as water solubility, UV barrier, ability to gel, and cost-effectiveness [5,6]. As more gelatin is used as the base material for capsule production, the amount of waste generated and the cost of waste disposal in the nutraceutical industry are increasing [4]. Service Pack Manufacturing Company Ltd., in Pathum Thani, Thailand reported that GCW discarded as byproducts or leftovers accounts for up to 1000 kg/month [7]. Importantly, these byproducts cannot be reused as capsules due to the loss of primary properties, including their protective qualities. However, utilizing GCW by converting it into high-value nutritious products could be worthwhile, because it contains approximately 74% protein, 6.29% lipid, and various minerals [8,9]. This strategy would not only reduce waste disposal expenses and minimize environmental impact but also support the achievement of the Sustainable Development Goal (SDG) of achieving zero waste by 2030. Unfortunately, the utilization of traditional forms of GCW is limited due to challenges such as transportation, oxidation, and the need for a large storage area. Hence, the transformation of GCW from its normal solid form to a powdered form would be beneficial.

Spray drying is the most widely used method in the food industry for producing dry powders. This technique is closely related to microencapsulation, wherein liquid formulations (solutions, suspensions, or emulsions, either aqueous or organic) are converted into stable powders from food matrices [10,11,12]. Spray drying offers several advantages, including being cost-effective and fast and requiring simple equipment [13,14]. One of the main factors affecting the properties of microencapsulated powders is closely related to the structural properties of the wall material [15]. Spray-dried gelatin without proper wall material can result in sticky products that adhere to the internal walls of the drying chamber [16]. Ideally, the wall material must have low viscosity, good solubility, ready availability, and biocompatibility, while existing in great diversity and having a low cost and low toxicity [15,17]. Maltodextrin is commonly used as a drying agent due to its numerous benefits, including high water solubility, low viscosity, and neutral color. It also helps regulate sugar content, minimize acidity and pH loss, control total dissolved solids, and enhance viscosity. Additionally, maltodextrin slows down crystallization, reduces stickiness and hygroscopicity, and improves the shelf-life stability of food products [12,13]. In addition, factors such as the feeding rate, inlet and outlet temperatures, initial solid concentration, surface tension, and intrinsic properties of the drying material affect the properties of gelatin [18]. Nevertheless, there have been no report studies on the characteristics and quality properties of GCW prepared using the spray-drying technique. Therefore, this study aimed to examine the impact of maltodextrin addition on the characteristics of GCWP produced through the spray-drying technique.

## 2. Materials and Methods

### 2.1. Materials

Fish gelatin manufactured by Lapi Gelatine S.p.A. (Empoli, Italy), derived from tilapia skin, had a gel strength of approximately 240 Bloom. Maltodextrin (food-grade) was purchased from SU Chem, Bangkok, Thailand. Food-grade ethanol was procured from the Excise Department, Bangkok, Thailand. Di-sodium hydrogen phosphate anhydrous, sodium tetraborate, sodium azide, hydrochloric acid (37%), and sodium hydroxide were procured from Power Tech Chemical, Bangkok, Thailand. Acetonitrile and methanol were procured from T.S. Inter Lab, Bangkok, Thailand, and o-phthaldialdehyde (OPA) was procured from Agilent Technologies, Santa Clara, CA, USA.

### 2.2. Collection and Preparation of Gelatin Capsule Waste

The gelatin capsule waste (GCW) was provided by Service Manufacturing Co., Ltd., Bangkok, Thailand. Samples were packed in a polyethylene bag and transported to the Department of Fishery Products, Faculty of Fisheries, Kasetsart University, Bangkok, Thailand. Upon arrival, the samples were washed with 95% ethanol three times to remove any oil components and unwanted debris. Each sample was placed in a polyethylene bag and stored at 4 °C until further analysis, but not for longer than for 1 month.

### 2.3. Spray Drying

The washed GCW was mixed with warm distilled water (50 °C) at a ratio of 1:14 (*w*/*v*) (based on preliminary results) and then gently mixed with maltodextrin, calculated based on the ratio of gelatin to maltodextrin (1:1, 1:2, 1:3, 1:4, and 1:5 (*w*/*w*)). Next, each mixture was heated in a water bath (WNB45; Memmert; Büchenbach, Germany) at 70 ± 2 °C for 20 min, with continuous stirring at 360 rpm using a mechanical overhead stirrer (RW28 Digital; IKA: Staufen, Germany). Subsequently, each mixture was spray-dried using a Mini Spray dryer (B-290; BUCHI; Labortechnik, Flawil, Switzerland). The operating conditions (determined based on preliminary results) were 190 °C inlet temperature and 120 °C outlet temperature, with 13% pump feed. Spray-dried GCW samples with the different maltodextrin levels (0, 1, 2, 3, 4, or 5%, *w*/*v*) were referred to as G-0M, GCW-1M, GCW-2M, GCW-3M, GCW-4M, and GCW-5M, respectively. The powder samples were packed in separate vacuum bags until further analysis. Commercial fish gelatin powder was used as the control. Notably, GCW without maltodextrin (G-0M) was not included further in this study because its sticky nature prevented it from being spray-dried.

### 2.4. Chemical Analysis

Proximate analysis including fat, protein, ash, and moisture contents was conducted based on the methods of Horwitz and Latimer [19]. Amino acid analysis was conducted as described by Phetchthumrongchai, et al. [20]. Each GCWP sample (0.5 g) was hydrolyzed with 6 M HCl (5 mL) or with 4.2 M NaOH (for tryptophan analysis) using a heating bath (B-300; Buchi, Flawil, Switzerland) at 110 °C for 24 h. Each hydrolyzed solution was diluted with 50 mL of distilled water (high-performance liquid chromatography grade), passed through a 0.22 μm nylon membrane filter, and transferred into a vial (1 μL) for injection. Amino acids were derivatized using OPA and analyzed based on high-performance liquid chromatography (1200 infinity series; Agilent; Palo Alto, CA, USA) equipped with a Poroshell-120 column (HPH-C18, 4.6 × 100 mm, 2.7 μm internal diameter). Mobile phase A consisted of 10 mM disodium hydrogen phosphate and 10 mM sodium tetraborate buffer, with the pH adjusted to 8.2 using 37% concentrated HCl and 5 mM sodium azide. Mobile phase B contained 45% acetonitrile, 45% methanol, and 10% deionized water (*v*/*v*/*v*). The analysis was performed at a flow rate of 1.0 mL/min, with the column temperature maintained at 40 °C and an injection volume of 1 µL. The fluorescent detector (FLD) was set to an excitation wavelength of 230 nm and an emission wavelength of 450 nm [21]. The elution gradient system was applied followed the Agilent Technologies protocol. The content of amino acids was reported as grams per 100 g protein.

### 2.5. Physico-Chemical Analysis

#### 2.5.1. Yield

The yield (%) of the gelatin capsule waste powder (GCWP) was calculated as described by Rather, et al. [22]. The yield was defined as the dry weight of the gelatin powder with maltodextrin relative to the initial dry weight of gelatin and maltodextrin, and it was calculated using the following equation:

$$\%\mathrm{Yield}=\frac{\mathrm{Weight}\;\mathrm{of}\;\mathrm{dry}\;\mathrm{powder}\;\mathrm{gelatin}\;\left(g\right)}{\mathrm{Initial}\;\mathrm{dry}\;\mathrm{weight}\;\mathrm{of}\;\mathrm{the}\;\mathrm{galtin}\;\mathrm{and}\;\mathrm{maltodextrin}\left(g\right)}\times 100$$

#### 2.5.2. Water Activity and pH

Water activity of the GCWP was measured in triplicate using an AQUALAB 4TE water activity meter (Decagon Devices, Pullman, WA, USA). For pH determination, 1 g of gelatin was dissolved in 100 mL of distilled water, heated to 60–70 °C, and subsequently cooled to 25 °C. The pH of this solution was then measured in triplicate using an OHAUS Starter 3100 pH meter (OHAUS, Newark, NJ, USA).

#### 2.5.3. Hygroscopicity and Syneresis

Hygroscopicity was determined according to Kou, et al. [23]. A GCWP sample (1 g) was placed in a desiccator, with the relative humidity controlled at 75.29% using a saturated sodium chloride solution. Each sample was accurately weighed every hour, with the hygroscopicity determined as grams of absorbed moisture per 100 g of dry matter (g/100 g). Further syneresis was calculated based on a modification of the method of [24]. The gelatin solutions (6.67%) were prepared by dissolving the various GCWP preparations in distilled water at 60–70 °C. Each gelatin solution (30 mL) was poured into 50 mL centrifuge tubes and the mass (m1) was recorded, followed by chilling at 4 °C for 18 h. Before remeasurement, each sample was equilibrated at room temperature for 3 h. Next, it was centrifuged at 4 °C, at a speed of 5000 rpm, for 10 min, using a refrigerated centrifuge (UNIVERSAL 32R; Hettich; Kirchlengern, Germany). After centrifugation, the water on top of the solution was wiped off, and care was taken not to touch the solution. The tube was weighed (m2). The syneresis was calculated using the following equation:

$$\%S\mathrm{yneresis}=\frac{m1-\;m2}{m1}\times 100$$

#### 2.5.4. Solubility

The solubility was calculated based on a modification of the method of Chuaychan and Benjakul [16]. Each GCWP sample (2 g) was mixed with distilled water (50 mL) and stirred on a hot plate for 15 min at room temperature. Next, the mixture was poured into 50 mL centrifuge tubes and centrifuged at 3600× *g* for 15 min at 25 °C using a refrigerated centrifuge (UNIVERSAL 32R; Hettich; Kirchlengern, Germany). The undissolved portion of the solution was separated using Whatman filter paper No. 1 and heated at 150 °C until dry. The solubility was calculated using the following equation:

$$\%\mathrm{Water}\;\mathrm{solubility}=\frac{\mathrm{dried}\;\mathrm{residue}\;\mathrm{weight}\;\left(g\right)}{\mathrm{sample}\;\mathrm{weight}\;\left(g\right)}\times 100$$

### 2.6. Rheological and Textural Analysis

#### 2.6.1. Texture Profiling

Gelatin solution (6.67%) was prepared by dissolving GCWP in distilled water at 60–70 °C, according to the method of Petcharat and Benjakul [25]. The gelatin solution was poured into ring molds (3 cm diameter) and chilled at 4 °C for 24 h. Next, the ring molds were removed and texture profile analysis (TPA) was carried out using a texture analyzer (TA.XT Plus; Stable Micro Systems, Godalming, Surrey, UK) and a cylindrical aluminum probe (P/50). The measurement mode was set to TPA, with a pre-test speed of 1 mm/s, a test speed of 5 mm/s, and a post-test speed of 5 mm/s, using 50% strain. The parameters determined were hardness, adhesiveness, springiness, cohesiveness, gumminess, and chewiness. Measurements were repeated 15 times.

#### 2.6.2. Gel Strength and DSC

Gel strength was measured using a TA.XT Plus texture analyzer (Stable Micro Systems, Godalming, Surrey, UK) equipped with a P0.5 cylindrical probe, following the method described by Petcharat and Benjakul [25]. A 6.67% gelatin solution was prepared and molded into ring molds prior to testing. The analyzer was operated in GMIA Gelatin Bloom mode with a pre-test speed of 1.5 mm/s, a test speed of 1 mm/s, and a post-test speed of 1 mm/s, with a 4 mm distance. Gel strength, expressed as Bloom value (grams), was measured in fifteen replicates. Differential scanning calorimetry (DSC) was performed according to the method outlined by Rather, et al. [22] using a Mettler Toledo DSC 1 STARe System (Mettler Toledo, Greifensee, Switzerland). Approximately 4 mg of each gelatin powder sample was heated from 0 to 250 °C at a heating rate of 5 °C/min.

#### 2.6.3. Gelling and Melting Temperatures

Gelling and melting temperatures were determined using a rheometer (MCR302; Anton Paar, Graz, Austria) following the method described by Petcharat and Benjakul [26]. Gelatin solutions (6.67%) were prepared by dissolving each GCWP preparation in distilled water at 60–70 °C. A 2.5 cm parallel plate geometry with a 1.0 mm gap was used. Each sample was loaded onto the rheometer, and measurements were conducted at a scan rate of 1 °C/min, a frequency of 1 Hz, and an oscillating strain of 1% during both cooling (60 to 5 °C) and heating (5 to 90 °C) cycles. The elastic modulus (G′) and loss modulus (G″) were recorded. Gelling and melting temperatures were identified as the temperature at which tan δ (G″/G′) reached 1 (or δ = 45°).

### 2.7. Techno-Functional Property

The foaming capacity and foaming stability of the GCWP were calculated as described by Rather, et al. [22]. Each gelatin powder sample (1 g) was mixed with distilled water (50 mL) at 70 °C. The gelatin solution was homogenized at 10,000× *g* for 15 min to develop foam. Next, the homogenized solution was poured into a measuring cylinder for determination of the foaming capacity and foaming stability, which were calculated using the following equations:

$$\%Foaming\;capacity=\frac{Foam\;value-Initial\;liquid\;volume}{Initial\;liquid\;volume}\times 100$$

$$\%Foaming\;stability=\frac{Foam\;value\;after\;15\;min-Initial\;liquid\;volume}{Initial\;liquid\;volume}\times 100$$

### 2.8. Optical Analysis

Color analysis of the GCWP was performed using a HunterLab UltraScan VIS colorimeter (HunterLab, Reston, VA, USA). Fifteen replicate measurements were taken, and the L* (lightness), a* (redness/greenness), and b* (yellowness/blueness) values were recorded. The FTIR analysis of each GCWP sample was conducted as described by Rather, et al. [22] using a Bruker Tensor 27 FT-IR spectrometer (Bruker, Germany) equipped with diamond crystal attenuated total reflectance (ATR). Absorbance spectra were obtained at 500–4000 cm^−1^ based on 4 cm^−1^ resolution and 32 scanning times. The spectra were analyzed with the OPUS program (version 6.5).

### 2.9. Structural Analysis by Scanning Electron Microscopy (SEM)

The microstructure of the GCWP was visualized using an SEM (Quanta400; FEI; Tokyo, Japan) at an accelerating voltage of 15 kV. Prior to visualization, each sample was mounted on a brass stub and sputter-coated with gold to make it conductive.

### 2.10. Statistical Analysis

A completely randomized design (CRD) was used and all experiments were carried out in triplicate (n = 3). Data analysis was performed using analysis of variance (one-way ANOVA) with the SPSS software package (SPSS 23.0 for Windows; SPSS Inc.; Chicago, IL, USA). The significance of the means was determined using Duncan’s test with a confidence level of 95% (*p* < 0.05).

## 3. Results and Discussion

The gelatin capsule waste without added maltodextrin (G-0M) could not be spray-dried due to nozzle clogging and was therefore excluded from further analysis. Throughout this paper, the following abbreviations are used: CFG (commercial fish gelatin), GCW-1M (gelatin:maltodextrin:water at a ratio of 1:1:14), GCW-2M (gelatin:maltodextrin:water at a ratio of 1:2:14), GCW-3M (gelatin:maltodextrin:water at a ratio of 1:3:14), GCW-4M (gelatin:maltodextrin:water at a ratio of 1:4:14), and GCW-5M (gelatin:maltodextrin:water at a ratio of 1:5:14).

### 3.1. Proximate Studies

The results of the proximate composition analysis of the GCWP samples containing maltodextrin at various ratios are shown in Table 1. The commercial fish gelatin (CFG) had the highest moisture content and protein levels, likely due to its derivation from pure fish gelatin. The moisture content of the GCWP decreased as the concentration of maltodextrin increased, which could be attributed to the higher total solid content, which reduced the amount of water available for evaporation [27]. The addition of maltodextrin in the GCW resulted in a decrease in the protein content, concomitant with an increase in the carbohydrate content, especially at the highest level of maltodextrin addition. Maltodextrin, a product of starch hydrolysis, is composed of D-glucose units linked by (1–4) glycosidic bonds; consequently, its incorporation increased the carbohydrate content [28]. This result was in agreement with Rizqiati, et al. [28], who stated that increasing the concentration of maltodextrin in powdered bovine colostrum kefir led to a higher carbohydrate content and a reduction in the product’s protein content.

The moisture content values (%) of the GCWP samples containing different amounts of maltodextrin are shown in Table 1. Moisture content is an important powder characteristic and is correlated with water activity, flowability, stickiness, drying efficiency, oxidation of bioactive agents, and microbial growth [29]. A good-quality powder product should have a low moisture content [17]. In the spray-dried product, the moisture content refers to the amount of water present in the material and is influenced by the microcapsule production process. The moisture content values of the GCWP samples containing maltodextrin were in the range 0.17–10.78, being lower than in the control sample, which suggested that maltodextrin could reduce the water content in the product [30]. The moisture content of food powder should be between 4% and 6% for long-term storage, while water activity values are expected to be below 0.6 to prevent microbial contamination [31]. The current results were consistent with [32], in which the authors noted that incorporating maltodextrin decreased the moisture content of sweet potato powder hydrolyzed using amylase. How, et al. [33] reported that a higher ratio of maltodextrin in the reconstituted fermented milk resulted in lower syneresis. Thus, adding the maltodextrin had a direct impact on the moisture content of the resulting powder.

The yield values (%) of the GCWP samples containing maltodextrin at various ratios are shown in Table 1. The undetectable yield of the control sample (CFG) was due to it being a commercial powder. The GCW-5M sample had the highest yield content (15.22%), followed by GCW-4M, GCW-3M, GCW-2M, and GCW-1M, respectively. The increase in yield for GCW-5M could be attributed to its higher maltodextrin content, which increased the total solids and resulted in a higher yield [30]. This result was consistent with the findings of Chuaychan and Benjakul [16], who reported that adding maltodextrin to gelatin products and hydrolyzed gelatin from the scales of golden goatfish increased the yield content. Millinia, et al. [17] reported that the yield of microencapsulated roselle (*Hibiscus sabdariffa* L.) powders increased as the maltodextrin-to-extract ratio increased. Therefore, the increase in yield content was affected by the increasing amount of maltodextrin.

### 3.2. Physico-Chemical Properties

#### 3.2.1. Water Activity and pH

The water activity values of the GCWP samples containing different amounts of maltodextrin were in the range 017–0.54 (Table 2). There was a slight decrease as the maltodextrin content increased (*p* < 0.05). This could be explained by the increased maltodextrin content leading to a reduction in water activity [34]. This result was in agreement with the findings of Koc, et al. [35], who observed that the water activity of spray-dried gac powders decreased as the concentration of maltodextrin increased. Water activity values between 0.20 and 0.40 ensure the stability of stored products by inhibiting microbial and biochemical activities, while also preventing coagulation during storage [36]. Therefore, all the powders produced in the current study were within the range considered optimal for the stability of powdered products. The pH values of the GCWP samples containing different amounts of maltodextrin are shown in Table 2.

The pH of the GCWP samples was in the range of 5.88–6.02 and increased significantly with higher maltodextrin content. The highest pH was observed in the GCW-5M sample. This increase in pH could be attributed to the maltodextrin compound containing many hydroxyl groups (OH), resulting in increased basicity of the gelatin powder [37]. This result was in agreement with the results of Caliskan and Nur Dirim [38], who reported that increasing the amount of maltodextrin produced a significant increase in the pH for sumac extract powders.

#### 3.2.2. Hygroscopicity

**Figure 1:**
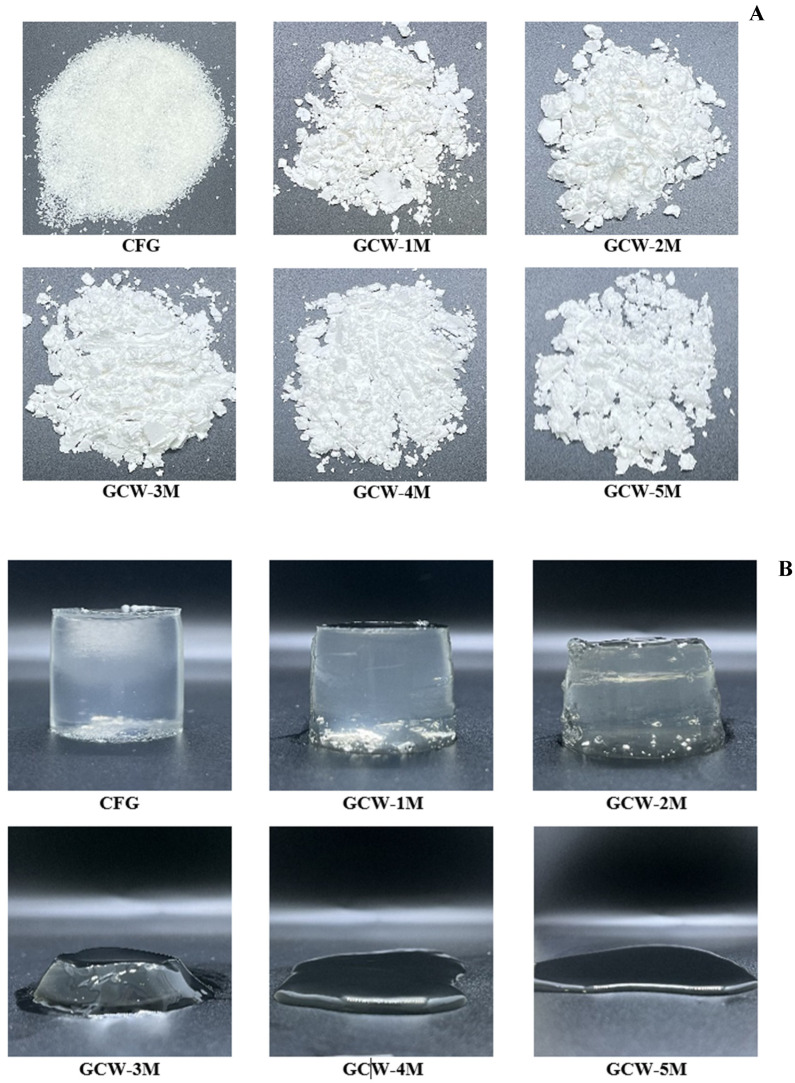
Appearance of colored gelatin capsule waste powder (**A**) and gelatin capsule waste gels (**B**), containing varying amounts of maltodextrin.

Generally, GCWP is highly hygroscopic, meaning that its spray-dried particles can readily absorb moisture from the surrounding air. However, if appropriate precautions are not taken, this can lead to the powder becoming sticky and causing caking [39,40]. In the current study, the hygroscopicity of the GCWP was in the range of 11–14% (Table 2 and Figure 1A), suggesting that it had low hygroscopicity. Comparable results were observed in chayote (*Sechium edule*) powder produced with fructans as a carrier [41]. In the current study, increasing the maltodextrin concentration resulted in decreased hygroscopicity of the GCWP, which could have been due to maltodextrin having low hygroscopicity, which reduces its ability to absorb moisture from the surrounding environment into the powder [42,43]. This was supported by Zhang, et al. [40], who reported that increasing the maltodextrin content could reduce the hygroscopicity of prebiotic xylooligosaccharide powder.

#### 3.2.3. Syneresis and Solubility

The syneresis values of the GCWP samples containing different amounts of maltodextrin are shown in Table 3. Syneresis is the phenomenon of liquid being exuded from a gel, which is basically undesirable for a gel product [44]. Based on the current results, the syneresis of the resulting powders was significantly higher than that of the control sample. The addition of maltodextrin may loosen the protein gel structure, resulting in high syneresis. This result was consistent with [45], who observed that the addition of maltodextrin to dry powder extract of *Satureja montana* decreased the water-holding capacity. Furthermore, a higher temperature during the spray-drying process most likely caused protein degradation, thereby producing protein fragments and reducing the water-holding capacity [46]. When maltodextrin was added at higher levels, there was no measurable syneresis result for the GCW-3M, GCW-4M, and GCW-5M samples, as they failed to form a gel during setting, as indicated in Figure 1B. Thus, the addition of maltodextrin altered the gelation properties of proteins, leading to increased syneresis.

The solubility values of the GCWP samples containing different amounts of maltodextrin are shown in Table 3. The solubility of gelatin is an essential property for its application in food systems [47]. The solubility values were in the range of 98.33–98.85%, with no significant differences compared to the control sample. The high solubility of the gelatin powder could be attributed to the water-soluble properties of the gelatin and maltodextrin, which are both hydrophilic substances that can form hydrogen bonds with water molecules [48,49]. This result was in agreement with Bebartta, et al. [50], who reported that using maltodextrin as a drying aid improved the solubility of protein isolates. Similar results were obtained in gelatins from spotted golden goatfish (Chuaychan and Benjakul [16]), with the gelatin-encapsulated maltodextrin having greater solubility. Thus, the solubility of the GCWP was influenced by the maltodextrin level.

#### 3.2.4. Amino Acid Composition

The amino acid components of the GCW sample containing 1% maltodextrin (GCW-1M) are shown in Table 4. In the current study, the major amino acids found in the control sample (CFG) were glycine (Gly), proline (Pro), hydroxyproline (Hypro), glutamic (Glu), and alanine (Ala). This was consistent with another study that reported Gly, Ala, Pro, and Hypro as the main amino acids in gelatins [51]. In general, the composition of amino acid gelatin is Gly-X-Y, where X is generally Pro and Y is generally Hypro [52]. The highest amounts of amino acids (Gly, Pro, and Hypro) were in the GCW-1M, which contained the main amino acids in gelatin. However, the amounts of these amino acids were lower than in the control, perhaps because the addition of supplemental ingredients during nutraceutical capsule production to achieve specific properties resulted in changes to the amino acid content. The lower amounts of proline and hydroxyproline were strongly related to the decrease in gel strength, as shown in Table 4. This finding is in agreement with the findings of [53]. Furthermore, the addition of maltodextrin, the partial thermal degradation of amino acids during spray drying, the Maillard reaction, and glycosylation reactions could have been co-factors altering the amino acid content. These results were consistent with Zhang, et al. [54], who reported that during the combined modification of soy protein with maltodextrin, two phenomena may occur: first, the proteins crosslink with maltodextrin to form high-molecular-weight SPI-Md conjugates; and then, these conjugates are hydrolyzed into smaller fractions and amino acids. Xue, et al. [55] reported that the lysine and arginine contents in soy protein isolate conjugated with polysaccharides (maltodextrin and gum acacia) decreased after processing using a dry-heated Maillard reaction. Therefore, the amount of maltodextrin and the drying process affected the amino acid content of GCWP.

### 3.3. Rheology and Textural Profiling

#### 3.3.1. Texture Profile Analysis and Gel Strength

The texture profile analysis results for the GCWP samples containing different amounts of maltodextrin are shown in Table 4 and Figure 1B. The highest hardness was found in the control sample, followed by the GCW-1M and GCW-2M. These results were correlated with gel strength. The addition of maltodextrin at higher levels to the gelatin capsule waste solution obstructed gel formation, as observed in samples GCW-3M, GCW-4M, and GCW-5M. This was attributed to the dilution effect of maltodextrin on gelatin, which might result from maltodextrin increasing the solid content of the gelatin solution and obstructing its gelling ability. Additionally, the addition of maltodextrin to gelatin solutions may interfere with the crosslinking of gelatin molecules, leading to a weaker gel or even preventing gel formation altogether [56]. The current result was in agreement with Bian, et al. [57], who reported that emulsion gel containing 15% maltodextrin and 25% gelatin had lower gel hardness than a gel made from 25% pure gelatin. Furthermore, it was assumed that high temperatures during spray drying could induce conformational changes in proteins, resulting in the loss of their gelation properties. Therefore, the amount of maltodextrin influenced the gelation process of gelatin.

#### 3.3.2. Rheological Behavior

**Figure 2:**
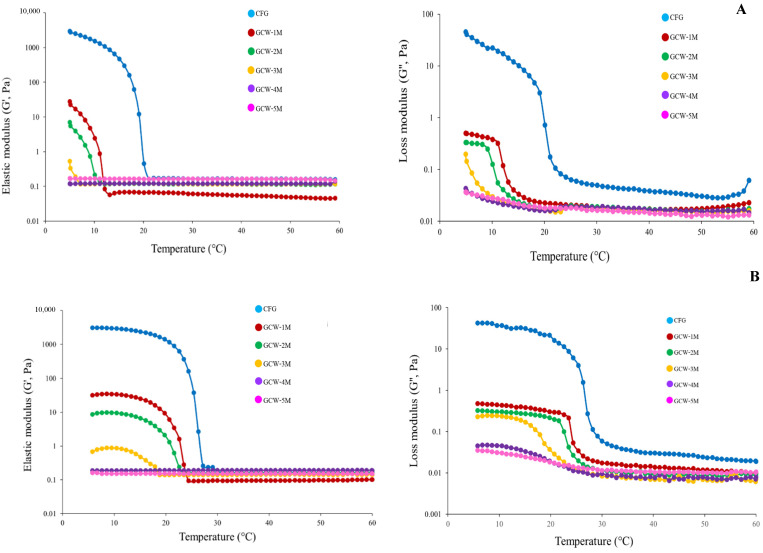
Elastic modulus (G′) and loss modulus (G″) of 6.67% gelatin capsule waste powder containing different amounts of maltodextrin during cooling from 60 °C to 5 °C (**A**) and from 5 °C to 60 °C (**B**).

The rheological behavior can indicate variations in the intermolecular forces of proteins, offering valuable insights into their physicochemical characteristics [58]. The G′ and G″ values of the GCWP samples containing different amounts of maltodextrin are presented in Figure 2. In general, G′ represents the energy stored as mechanical energy following the application of a deforming force and reflects variations in the sample’s elasticity during the gelation process [25,59], while G″ represents the gel’s viscous properties [60]. The curves of both these parameters displayed similar patterns, with the G′ and G″ values of the samples gradually decreasing as the temperature increased, indicating a step-by-step conversion from a solid-like gel to a liquid-like solution [61]. Furthermore, the control had greater values for the storage modulus (G′) and loss modulus (G″) than the other samples. This result was consistent with the control sample having the highest gel strength (Table 4). The addition of higher levels of maltodextrin decreased the G′ and G″ values, indicating a liquid-like consistency. This may have been due to the maltodextrin interfering with the ability of the gelatin to form a strong gel. Zhao, et al. [62] demonstrated that glycosylation and non-glycosylation modifications can alter the molecular structure and influence the strength of intramolecular chemical bonds in gelatin molecules. In addition, the current result was similar to the findings of Sinthusamran, et al. [63], who reported that the Gʹ value of fish gelatin containing κ-carrageenan gel decreased as the κ-carrageenan content increased. Thus, maltodextrin seemed to interfere with gel formation in fish GCWP.

#### 3.3.3. Differential Scanning Calorimetry

**Figure 3:**
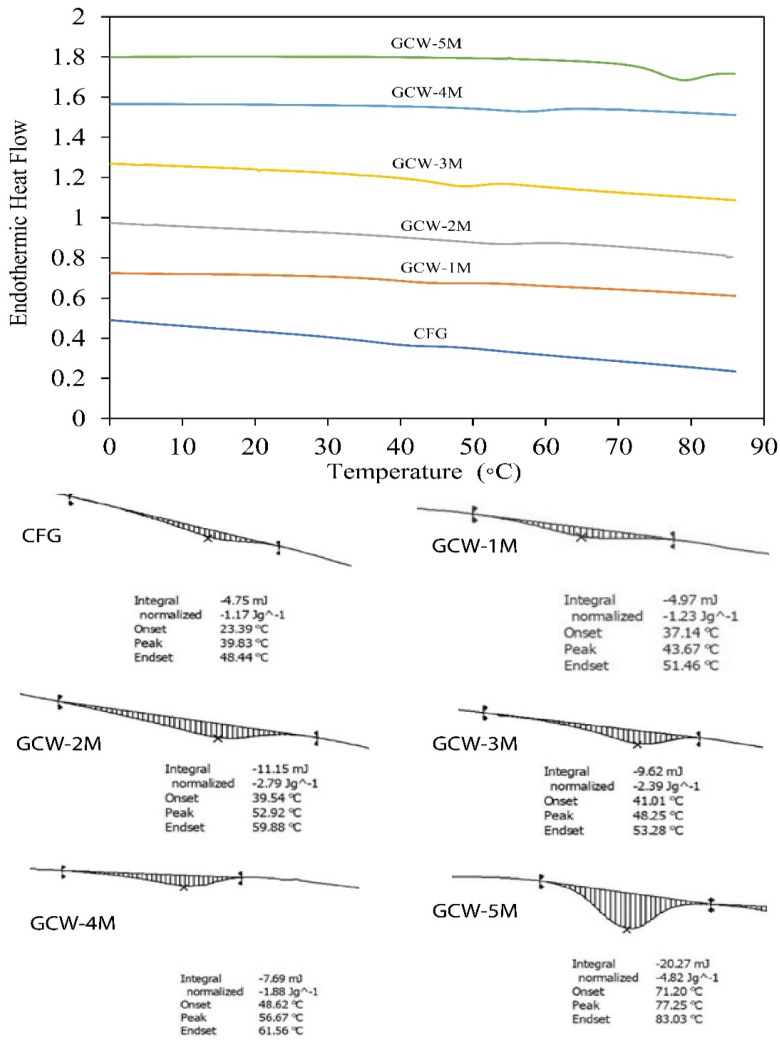
Differential scanning calorimetry (DSC) analysis of thermal changes in gelatin capsule waste powder containing different amounts of maltodextrin.

The DSC thermograms of the GCWP samples containing different amounts of maltodextrin are shown in Figure 3. The Tg value denotes the temperature at which the material transitions from an amorphous state to a rubbery and viscous state [64]. Furthermore, the Tg value of spray-dried powder serves as a key indicator of its stability during extended storage, as it is widely recognized that maintaining spray-dried products below their glass transition temperature enhances stability [65]. In the current study, the Tg value of the control gelatin (CFG) was 23.39 °C. Notably, as the maltodextrin ratio increased, the Tg of all the treatment samples gradually increased. The shift of the Tg peaks to higher values in the treatments indicated an interaction between maltodextrin and the GCW, resulting in changes in physical properties. In general, Tg is influenced by factors such as the material’s moisture content, chemical composition, and molecular weight [65]. Stępień and Witczak [66] reported that the addition of maltodextrin contributed to an increase in the Tg value of green pea (*Pisum sativum* L.) powder. Thus, the DSC analysis demonstrated that the thermal behavior of gelatin can be greatly modified by the addition of maltodextrin.

### 3.4. Techno-Functional Properties

The foaming capacity and foaming stability values of the GCWP samples containing different amounts of maltodextrin are shown in Table 4. The foaming capacity refers to how well proteins can form a layer around air bubbles in a liquid, which mainly depends on how the protein molecules move, unfold, and arrange themselves at the air–water surface [67]. There was significantly lower foaming capacity in the treatments than in the control sample, which might have resulted from the composition of the GCWPs, which contained not only gelatin but also other substances, such as oil and active ingredients, which might have impeded the protein’s foam-forming ability. The addition of maltodextrin at higher levels tended to improve the foaming capacity and stability, as maltodextrin serves as a foam stabilizer [68]. It is possible that there was some interaction within the maltodextrin–protein complex in the bulk, which increased the particle diffusion speed to the bubble interface, making the adsorption higher and influencing the increase in foaming capacity. Khatri, et al. [69] reported that a higher maltodextrin concentration enhanced the foaming stability of black mulberry juice powder obtained using a foaming process. Thus, the addition of maltodextrin affected the foaming capacity and stability of GCWP.

### 3.5. Color and FTIR Profiling

The color parameters (L*, a*, b*) of the GCWP samples containing different amounts of maltodextrin are presented in Table 2. Color is a key quality factor, as it indicates both the sensory appeal and the overall quality of powders [38]. In the current study, the samples with added maltodextrin had higher L* values than the control sample (CFG), regardless of the maltodextrin levels. Conversely, lower a* and b* values were noted when maltodextrin was added, suggesting that the white color of the maltodextrin enhanced the brightness (L*) and reduced the a* and b* values of the powder [70]. Consequently, the resulting powder likely had higher whiteness and less yellowness. This result was similar to the findings of Wartini, et al. [71], who stated that the brightness level increased with the incorporation of a high content of maltodextrin. Caliskan and Nur Dirim [38] reported that as the maltodextrin concentration increased, the L* values increased, while the a* and b* values of sumac extract powders decreased. Therefore, the maltodextrin influenced the coloration of the gelatin powders, particularly through the natural color of the incorporated hydrocolloid.

**Figure 4:**
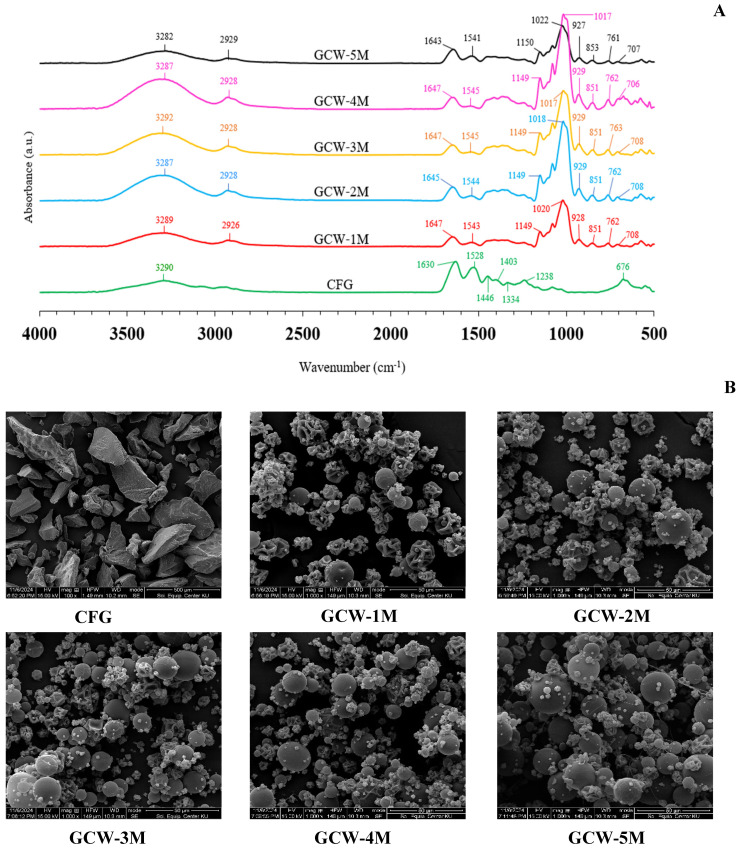
(**A**) FTIR spectra and (**B**) microstructure images of gelatin capsule waste powder with varying amounts of maltodextrin. Remark: The magnification for the control sample was 500×, whereas for the treatment samples, it was 1000×. The control sample was presented at 500× because, at 1000× magnification, the overall shape of the powder crystals was not clearly visible due to their larger size.

Commonly, FTIR spectroscopy is used to investigate the functional groups and structure of substances [72]. The infrared spectra of the GCWP samples containing different amounts of maltodextrin are shown in Figure 4A. For the CFG sample, the major peaks were amide A, B, I, II, and III [58,73]. The presence of these bands arises from the vibrational motions of peptide bonds that link together the amino acids that constitute the protein, in this case the gelatin [74]. The amide I peak, which appears in the range of 1600–1700 cm^−1^, corresponds primarily to C=O stretching vibrations, which are associated with the secondary structure of the protein, particularly the carbonyl stretch in the peptide bond. The amide II band, located between 1300 and 1600 cm^−1^, is primarily related to N-H bending coupled with C-N stretching vibrations [75]. The amide III band, observed in the 1200–1300 cm^−1^ range, is due to C-N stretching vibrations coupled with N-H in-plane bending vibrations, as well as contributions from C-C stretching [74]. The spectral region in the range of 3400–3440 cm^−1^ corresponded to the band of amide A, whereas amide B corresponded to the asymmetric stretching vibration of =C-H and -NH^3+^ [75]. The presence of maltodextrin in the samples was indicated by the major peak in the wavenumber range of 700–800 cm^−1^. The wavenumber absorption band in the range of 800–1300 cm^−1^ has been considered the “carbohydrate region” and is characterized by sharp overlapping peaks resulting from glycosidic linkages consisting of CO stretching, CC stretching, and COH bending vibrations [72]. With increasing maltodextrin concentration in the samples, there was an appearance of peak intensity at 1017–1022 cm^−1^, which is associated with the carbohydrate region. Kutzli, et al. [76] reported that the major band at around 1000 cm^−1^ was a combination of the vibration of the C-C pyranoid ring of the glucose monomers of maltodextrin, the vibration of the C-O-C glycosidic bond, and the stretching of the C-OH side group. The treatment samples with maltodextrin also showed an increased intensity of the band at 3282–3292 cm^−1^, which can be attributed to the strengthening of hydrogen bonds. This enhancement of hydrogen bonding may be due to electrostatic and/or hydrophilic interactions between gelatin and maltodextrin, which could facilitate the formation of additional hydrogen bonds in the gelatin matrix [77]. Vargas-Muñoz and Kurozawa [78] reported that the maltodextrin spectrum showed bands at 3395 cm^−1^ (O-H stretching), 2932 cm^−1^ (C-H stretching), 1638 cm^−1^ (H_2_O absorbed in amorphous region), 1417 cm^−1^ (CH_2_ bending), 1154 and 1079 cm^−1^ (C-O-H bending and C-O stretching, which are typical of carbohydrates), 1025 cm^−1^ (angular deformation of =CH and =CH_2_ bonds, carbohydrates peak), 931 and 857 cm^−1^ (deformation of CH_2_ and C_1_-H), and 762 and 711 cm^−1^ (structural condition of the pyranose ring). The complete disappearance or fading of the peak at 1238, 1344, 1403, and 1446 cm^−1^ in the control sample, along with the appearance of peaks at 1018–1022, 1149–1150, and 2925–2926 cm^−1^ in the treated samples, might be due to covalent cross-linking and glycosylation. These changes could arise from interactions between the gelatin and the added maltodextrin during the treatment process. Glycosylation is the process of attaching carbohydrate molecules, such as those in maltodextrin, to proteins like gelatin. This interaction is typically due to the formation of covalent bonds between the hydroxyl groups of the carbohydrate and the amino acids in the protein, particularly the amine or hydroxyl groups [79]. In the case of gelatin, which is a protein derived from collagen, the hydroxyl groups from the carbohydrate (maltodextrin) can react with the amino groups of the protein to form glycosidic bonds or other covalent linkages, leading to glycosylation. These results corresponded with the findings of Geng, et al. [80], who reported that the glycosylation of fish gelatin with κ-carrageenan (κC), high methoxyl pectin (HMP), and d-galactose (Gal) could lead to changes in the FTIR spectra. Therefore, the addition of maltodextrin influenced the functional groups of GCWP.

### 3.6. Morphological Studies

The morphology of the control and the GCWP samples was determined using SEM, with the SEM images for all the samples being presented in Figure 4B. The SEM micrographs demonstrated that the microcapsules displayed primarily spherical and semi-spherical shapes with considerably dented surfaces, even though, typically, spray-dried particles are expected to have a uniformly spherical structure. The dented surfaces of the spray-dried particles were attributed to particle shrinkage occurring during the drying process [81]. At a lower maltodextrin ratio (GCW-1M), many dented surfaces were observed, perhaps because the gelatin particles were not completely coated with maltodextrin. Consequently, when the liquid material was sprayed through the nozzle, the hot air passing through the drying chamber took longer to fully dry the liquid material, resulting in a surface that was not smooth. These results were consistent with the finding, by Achmad Kosasih, et al. [13], that incompletely coated gelatin particles with maltodextrin had irregular shapes. As the maltodextrin concentration increased, the particles tended to be more spherical and more evenly dispersed. Similar results were reported by Zhang, et al. [40] regarding the spray-drying of prebiotic xylooligosaccharides (XOS). Thus, the maltodextrin levels directly affected the characteristics of the GCWP.

This study successfully demonstrated the production of gelatin capsule waste powder (GCWP) using maltodextrin and spray-drying techniques, showcasing its potential as a functional food ingredient. The resulting GCWP exhibited improved techno-functional and physico-chemical properties, including a non-sticky texture, increased yield, enhanced foaming capacity, and improved foam stability compared to the original GCW. The incorporation of maltodextrin played a crucial role in stabilizing the drying process, reducing stickiness, and improving the overall handling and storage properties of the powder.

Furthermore, the GCWP’s composition, particularly its rich amino acid profile, suggests its potential use in various food and feed applications. As a functional food ingredient, GCWP could be incorporated into high-protein snacks, bakery products, and sports nutrition formulations, in which its emulsification and gelation properties may contribute to texture and stability improvements. Future research should investigate methods to further enhance its gel properties, as well as assessing the long-term storage stability of spray-dried GCWP to ensure its viability in commercial applications.

## Figures and Tables

**Table 1:** Proximate analysis and yield of gelatin capsule waste powder containing different amounts of maltodextrin.

Sample	Moisture (%)	Fat (%)	Ash (%)	Protein (%)	Carbohydrate (%)	Yield
CFG	10.78 ± 0.14 ^a^	0.03 ± 0.02 ^b^	0.04 ± 0.03 ^c^	82.35 ± 0.52 ^a^	6.80 ± 0.45 ^f^	-
GCW-1M	3.02 ± 1.27 ^b^	0.06 ± 0.03 ^ab^	0.13 ± 0.07 ^a^	26.15 ± 0.10 ^b^	70.64 ± 1.36 ^e^	10.06
GCW-2M	0.85 ± 0.16 ^c^	0.03 ± 0.02 ^b^	0.11 ± 0.02 ^ab^	15.87 ± 0.03 ^c^	83.13 ± 0.19 ^d^	11.38
GCW-3M	0.29 ± 0.21 ^c^	0.03 ± 0.01 ^b^	0.06 ± 0.03 ^bc^	11.30 ± 0.06 ^d^	88.33 ± 0.29 ^c^	14.67
GCW-4M	0.17 ± 0.09 ^c^	0.12 ± 0.09 ^a^	0.04 ± 0.03 ^c^	9.39 ± 0.16 ^e^	90.28 ± 0.31 ^b^	31.91
GCW-5M	0.21 ± 0.16 ^c^	0.08 ± 0.01 ^ab^	0.03 ± 0.02 ^c^	7.71 ± 0.00 ^f^	91.96 ± 0.10 ^a^	41.70

Note: Values (mean ± standard deviation) with different lowercase superscripts in same column are significantly different (*p* < 0.05).

**Table 2:** Water activity (a_w_), pH, hygroscopicity, and color values of gelatin capsule waste powder containing different amounts of maltodextrin.

Sample	a_w_	pH	Hygroscopicity (%)	L*	a*	b*
CFG	0.54 ± 0.00 ^a^	5.41 ± 0.04 ^d^	13.74 ± 0.11 ^b^	86.33 ± 1.50 ^c^	−0.54 ± 0.21 ^b^	9.85 ± 1.06 ^a^
GCW-1M	0.20 ± 0.00 ^b^	5.88 ± 0.05 ^c^	13.94 ± 1.24 ^b^	95.91 ± 0.32 ^b^	−0.24 ± 0.03 ^a^	3.82 ± 0.20 ^b^
GCW-2M	0.17 ± 0.01 ^c^	5.91 ± 0.01 ^bc^	13.89 ± 1.74 ^b^	96.36 ± 0.19 ^ab^	−0.23 ± 0.05 ^a^	2.80 ± 0.09 ^c^
GCW-3M	0.14 ± 0.01 ^de^	5.95 ± 0.03 ^b^	13.88 ± 0.67 ^b^	96.65 ± 0.23 ^a^	−0.22 ± 0.02 ^a^	2.59 ± 0.10 ^cd^
GCW-4M	0.15 ± 0.01 ^d^	6.01 ± 0.01 ^a^	13.40 ± 1.15 ^b^	96.71 ± 0.28 ^a^	−0.17 ± 0.04 ^a^	2.23 ± 0.09 ^d^
GCW-5M	0.14 ± 0.01 ^e^	6.02 ± 0.01 ^a^	11.18 ± 1.09 ^a^	96.57 ± 0.21 ^a^	−0.24 ± 0.03 ^a^	2.50 ± 0.13 ^cd^

Note: Values (mean ± standard deviation) with different lowercase superscripts in same column are significantly different (*p* < 0.05).

**Table 3:** Texture profile analysis, gel strength, syneresis, solubility, foaming capacity, and foaming stability of gelatin capsule waste powder containing different amounts of maltodextrin.

Samples	Texture Profile Analysis					Gel Strength (g)	Syneresis (%)	Solubility (%)	Foaming Capacity (%)	Foaming Stability (%)
	Hardness (N)	Adhesiveness (N × s)	Springiness (cm)	Gumminess (N)	Chewiness (N × cm)					
CFG	11.50 ± 1.39 ^a^	−121.20 ± 79.41 ^b^	0.94 ± 0.03 ^ab^	10.65 ± 1.23 ^a^	10.03 ± 1.16 ^a^	146.25 ± 16.85 ^a^	0.12 ± 0.02 ^a^	98.37 ± 0.71 ^a^	32.00 ± 10.58 ^a^	28.67 ± 11.37 ^a^
GCW-1M	1.85 ± 0.25 ^b^	−8.12 ± 7.31 ^a^	1.42 ± 1.06 ^a^	1.13 ± 0.33 ^b^	1.56 ± 1.09 ^b^	17.22 ± 0.38 ^b^	0.19 ± 0.07 ^ab^	98.74 ± 0.61 ^a^	5.33 ± 1.15 ^c^	0.67 ± 1.15 ^c^
GCW-2M	1.77 ± 0.56 ^b^	−10.30 ± 0.32 ^a^	0.46 ± 0.01 ^b^	0.36 ± 0.00 ^b^	0.17 ± 0.00 ^c^	15.14 ± 0.31 ^b^	0.21 ± 0.02 ^b^	98.85 ± 0.05 ^a^	8.00 ± 2.00 ^c^	4.00 ± 2.00 ^bc^
GCW-3M	ND	ND	ND	ND	ND	ND	ND	98.55 ± 0.25 ^a^	8.67 ± 2.31 ^c^	5.33 ± 3.06 ^bc^
GCW-4M	ND	ND	ND	ND	ND	ND	ND	98.58 ± 0.07 ^a^	17.33 ± 1.15 ^b^	11.33 ± 1.15 ^b^
GCW-5M	ND	ND	ND	ND	ND	ND	ND	98.33 ± 0.14 ^a^	20.00 ± 0.00 ^b^	10.67 ± 1.15 ^b^

Note: Values (mean ± standard deviation) with different lowercase superscripts in same column are significantly different (*p* < 0.05), ND: not detected.

**Table 4:** Amino acid components of GCWP containing different amounts of maltodextrin.

Amino Acid Component (g/100 g Protein)	Abbrevations	Treatment	
		CFG	GCW-1M
Alanine	Ala	7.34 ± 0.01 ^a^	0.90 ± 0.84 ^b^
Arginine	Arg	4.63 ± 0.01 ^a^	0.56 ± 0.51 ^b^
Aspartic acid	Asp	3.08 ± 0.01 ^a^	0.84 ± 0.00 ^b^
Cysteine	Cys	0.02 ± 0.00 ^b^	0.81 ± 0.81 ^a^
Glutamic acid	Glu	7.31 ± 0.01 ^a^	1.91 ± 0.00 ^b^
Glycine	Gly	16.90 ± 0.01 ^a^	4.49 ± 0.00 ^b^
Histidine	His	0.32 ± 0.02 ^a^	0.17 ± 0.09 ^b^
Hydroxy proline	Hypro	7.52 ± 0.04 ^a^	2.27 ± 0.00 ^b^
Isoleucine	Ile	0.96 ± 0.00 ^a^	0.46 ± 0.19 ^b^
Leucine	Lue	2.02 ± 0.00 ^a^	0.27 ± 0.27 ^b^
Lysine	Lys	1.96 ± 0.00 ^a^	0.29 ± 0.21 ^b^
Methionine	Met	0.85 ± 0.01 ^b^	1.19 ± 1.11 ^a^
Phenylalanine	Phe	1.41 ± 0.00 ^a^	0.17 ± 0.17 ^b^
Proline	Pro	9.01 ± 0.05 ^a^	2.49 ± 0.00 ^b^
Serine	Ser	2.54 ± 0.01 ^a^	0.66 ± 0.00 ^b^
Threonine	Thr	2.01 ± 0.01 ^a^	0.39 ± 0.00 ^b^
Tryptophan	Trp	0.00 ± 0.00 ^b^	0.85 ± 0.85 ^a^
Tyrosine	Tyr	0.30 ± 0.00 ^b^	0.98 ± 0.91 ^a^
Valine	Val	1.64 ± 0.00 ^a^	0.25 ± 0.17 ^b^

Note: (CFG) commercial fish gelatin, (GCW-1M) gelatin:maltodextrin:water 1:1:14, values (mean ± standard deviation) with different lowercase superscripts in same row are significantly different (*p* < 0.05).

## Data Availability

The original contributions presented in the study are included in the article, further inquiries can be directed to the corresponding authors.
